# The Money Issue

**DOI:** 10.1371/journal.pmed.0030073

**Published:** 2006-01-31

**Authors:** Mpho Selemogo

**Affiliations:** **1**University of MelbourneMelbourneAustralia

Auvert et al. must be commended for showing some appreciation of the ethical issues raised by their research trial [ 1]. The Research Article itself and the accompanying ethical review by Cleaton-Jones [ 2], however, curiously seem to take the money issue lightly. The *PLoS Medicine* Editorial is quite right in identifying the R300 payment to participants as an issue [ 3].

Rather than just identifying what R300 means in terms of the euro, we need an idea of the sum's effect on the average person enrolled in the study in order to best review issues of autonomy, which are often so problematic in such research. What was its impact on the recruitment process? Was the average income for the participants so low that declining to participate in the study and turning down the money was not an economically feasible option? The absence of such critical socioeconomic data leaves us wondering if this money was meant as a force for recruitment or indeed as a compensation for participation, as the authors assert.
